# Analysis of benzodiazepine withdrawal program managed by primary care nurses in Spain

**DOI:** 10.1186/1756-0500-5-684

**Published:** 2012-12-13

**Authors:** Cristina Lopez-Peig, Xavier Mundet, Bartomeu Casabella, Jose Luis del Val, David Lacasta, Eduard Diogene

**Affiliations:** 1 Primary Care Center Raval Sud. Institut Català de la Salut, 08001, Barcelona, Spain; 2 Unitat de Suport de la Recerca Barcelona Ciutat - IDIAP Jordi Gol, redIAPP Institut Català de la Salut, Sardenya 375, 08025, Barcelona, Spain; 3 Universitat Autònoma de Barcelona, 08193, Bellaterra, Spain; 4 Primary Care Center La Salut. Institut Català de la Salut, 08914, Badalona, Spain; 5 Institut Català de Farmacologia, Barcelona, Spain; 6 Woodberry Practice. Winchmore Hill. Enfield. NHS trust, London, UK

**Keywords:** Benzodiazepine, Habituation drug therapy, Primary care

## Abstract

**Background:**

Benzodiazepine (BZD), the long-term treatment of which is harmful for cognitive function, is widely prescribed by General Practitioners in Spain. Based on studies performed in other countries we designed a nurse-led BZD withdrawal program adapted to Spanish Primary Care working conditions.

**Results:**

A pseudo-experimental (before-after) study took place in two Primary Care Centres in Barcelona. From a sample of 1150 patients, 79 were identified. They were over 44 years old and had been daily users of BZD for a period exceeding six months. Out of the target group 51 patients agreed to participate. BZD dosage was reduced every 2-4 weeks by 25% of the initial dose with the optional support of Hydroxyzine or Valerian. The rating measurements were: reduction of BZD prescription, demographic variables, the Short-Form Health Survey (SF-12) to measure quality of life, the Medical Outcomes Study (MOS) Sleep Scale, and the Goldberg Depression and Anxiety Scale.

By the end of the six-month intervention, 80.4% of the patients had discontinued BZD and 64% maintained abstinence at one year. An improvement in all parameters of the Goldberg scale (p <0.05) and in the mental component of SF-12 at 3.3 points (p = 0.024), as well as in most components of the MOS scale, was observed in the group that had discontinued BZD. No significant differences in these scales before and after the intervention were observed in the group that had not discontinued.

**Conclusions:**

At one year approximately 2/3 of the patients had ceased taking BZD. They showed an overall improvement in depression and anxiety scales, and in the mental component of the quality of life scale. There was no apparent reduction in the sleep quality indicators in most of the analysed components. Nurses in a Primary Care setting can successfully implement a BZD withdrawal program.

## Background

Long-term treatment with Benzodiazepine (BZD) is harmful at varying cognitive levels [1]. Even though there is a clear association between the use of BZD by the elderly and an increased risk of falls, fractures, and road traffic accidents BZD is still widely prescribed by General Practitioners in Spain [2-4]. In fact, BZD is taken by up to 10% of the population in most developed countries [5].

Time constraints and a general lack of motivation in dealing with mental health issues on the part of General Practitioners have contributed significantly to the elevated and inappropriate use of BZD in Primary Care [6]. Our study attempted to address this issue by giving nursing professionals (as opposed to General Practitioners) the leading role in managing BZD withdrawal programmes. Our final objective was to achieve a nurse-led success rate for the programme equivalent to that obtained by General Practitioners.

Due to the fact that the number of studies carried out in Spain focusing on BZD withdrawal is very limited, our guidelines were based on strategies employed by General Practitioners in other countries. After reviewing the literature on BZD withdrawal programs, and using the study by Curran et al. in the United Kingdom as a main reference [7], we designed a programme to be managed by nursing professionals in Spanish Primary Care. Our aim was to demonstrate that (a) those patients who volunteered to participate would cease BZD consumption after six months, and that abstinence would be maintained for at least a further six months (twelve months in total), and (b) reduction of BZD was not harmful for the patient.

## Methods

### Study design

This is a before-after pseudo experimental study conducted in two large Primary Care Centres based in Barcelona (Spain). It was approved by the local Institut Català de la Salut-Barcelona ethics committee.

### Participants

#### Nurses

Five qualified Primary Care nurses with varying levels of clinical experience agreed to participate. They were given training with respect to BZD usage and withdrawal programs in two 2-hour seminars which covered the following main topics: “Normal Sleep Physiology”, “Sleep Hygiene and Insomnia”, “Benzodiazepine History”, and “Models and Therapies for Withdrawal Programs”. The nurses were trained in assessing patients’ needs for pharmacological support so they could choose, at their clinical discretion, to give either a prescription of Hydroxyzine (25 mg per day) or advice on using over-the-counter Valerian products which have good tolerance, low risk, and reduced cost. Finally, they were provided with a thorough explanation of our protocol.

#### Patients

We identified patients of both sexes, over 44 years of age, who had used BZD daily for a period of more than 6 months. Participation was voluntary and written informed consent was obtained. Exclusion criteria were: epilepsy, cognitive impairment or dementia, hearing loss, severe visual defects, palliative care, and severe psychiatric illness. In addition, patients whose General Practitioner considered that their BZD withdrawal would be not be clinically appropriate were excluded.

### Protocol and baseline measures

In a similar manner to Curran et al. we assessed several functions, including quality of life and sleep. We additionally employed The Goldberg Scale for Depression and Anxiety to better understand BZD usage. Patients who met the inclusion criteria were informed by their doctor about the study and the benefits of ceasing BZD. If they agreed to participate they were asked to complete a socio-demographic data collection questionnaire. Their responses were then evaluated by nurses who conducted a semi-structured interview assessing their attitudes and beliefs about BZD and sleep. In addition to gathering administrative data from the questionnaire, the nurses described the risks and benefits of long term BZD consumption, side effects, and possible dependence. They also briefly explained the programme and the implications of participating in the study. Three self-administered questionnaires were given to patients to be completed at home: The Short-Form Health Survey (SF-12) to measure quality of life [8,9], the Medical Outcomes Study (MOS) sleep scale [10], and the Goldberg Depression and Anxiety scale [11]. Finally, a date was fixed to commence the withdrawal program. The general outline of the intervention program and each visit is summarized in Table 1.

**Table 1:** Intervention and data collection

	
**Visit Selection**	**Selection of candidates for study**
	**Patient**: Completes questionnaire.
	**Doctor**: Checks that patient meets criteria for study (reviews results of data collection questionnaire + nurse interview).
	**Nurse**: Semi-structured interview with patient. Consent obtained from patient.
	**If patient accepts**: Questionnaires / test (T) given to patient + set up of start date (VISIT 0)
**Visit 0**	**Collect questionnaires / tests + Brief educational intervention (BEI) + detoxification regimen given to patient**
**Visit 4**	**BEI + detoxification regimen plan given**
**Visit 8**	**BEI + detoxification regimen + Questionnaires / test (T) given to patient**
**Visit 12**	**BEI + Collect questionnaires / tests and new Questionnaires / test (T) given to patient**
**Visit 24**	**BEI + Collect questionnaires / tests (T)**

BEI: Brief educational Intervention.

T: test. Include: SFHF 12, Goldberg, and MOS.

Patients were evaluated by nurses every four weeks for the first 12 weeks. An assessment with the Goldberg Depression and Anxiety Scale, the quality of life scale (SF-12), and MOS sleep scale was performed at the initial visit, and at 12 and 24 weeks.

With regard to medication cessation, the baseline was the same in all cases. Patients could choose to continue taking either the same BZD or the equivalent dose of Diazepam which was then reduced by 25% every 2-4 weeks. If, according to the nurse’s assessment, pharmacological support with Hydroxyzine (25 mg per day) or Valerian was needed, the doctor would then be consulted. The doctor’s supervisory role was to review the nurse’s clinical decision, check the medication plan, and issue a prescription, if appropriate. The nurses also had the opportunity to provide feedback to the doctor. Information was extracted from patients’ notes, surveys, and questionnaires. The primary endpoint (no consumption of BZD after six and twelve months) variable was obtained verbally from the patient and confirmed by prescription data.

### Study outcome measures

The following variables were collected: socio demographic, toxic habits, antidepressant consumption, co-morbidity, use of BZD, and data from the assessment scales: the Goldberg Depression and Anxiety Scale, The Short-Form Health Survey (SF-12), and The Medical Outcomes Study (MOS) sleep scale.

### Statistical analysis

A data paired sample was calculated. Taking as a reference the results from various studies, a minimum BZD cessation of 20% was considered clinically relevant. Therefore, 40 patients (alpha error of 0.05 and beta error of 0.2) were required to give a statistically significant result. Mean with SDs and proportions were calculated for all variables. The Chi-square test was used to compare categorical data, and an unpaired Student t test was used to compare continuous variables. A two-tailed value of P<0.05 was considered statistically significant. Cohen’s d measured the effect size and compared it across the different variables.

## Results

**Figure 1:**
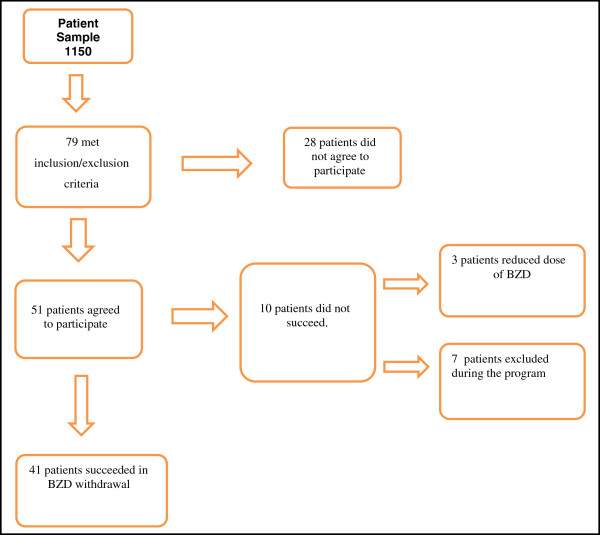
Flow chart describing the progression of participants during the study.

1150 patients were attended by their Primary Care doctors over an inclusion period of 3 months. From the 79 patients who met the inclusion criteria, 51 agreed to participate in the study (36 females and 15 males) (Figure 1). The mean age was 70.4 years (95% CI: 66.4 to 73.9 years). Socio-demographic characteristics are presented in Table 2.

**Table 2:** Patients’ features

**Features**	**N - (%)**
Natives or residents for more than 5 years	49 (96.1)
**Education:**	
Primary school	14 (27.5)
Secondary school	34 (66.7)
University or postgraduate.	3 (5.9)
**Employment situation:**	
Pensioners	36 (70.6)
Housewives	6 (11.8)
Active workers	9 (17.6)
**Consumption of drugs:**	
Did not use antidepressants	42 (82)
Consumed antidepressants:	9 (18)
a) SSRI	7 (14)
b) MAO	1 (2)
c)Tricycle Antidepressants	1 (2)
**Chronic disease:**	
One or more chronic diseases	43 (84.3)
COPD	10 (19.6)
Osteoarthritis	6 (11.8)
**Toxic habits:**	
Alcohol	2 (3.9)
Smoking	13 (25.5)
Other drugs	1 ( 2)
**Cause of BZD consumption**	
Sleep disorder	25 (0.5)
Anxiety	9 (0.2)
Depression	5 (0.1)
Other diagnoses	11 (0.2)

SSRI: Selective Serotonin Reuptake Inhibitor.

MAOI: Monoamine oxidase Inhibitors.

COPD: Chronic Obstructive Pulmonary Disease.

Regarding long term consumption, 22 patients (43.1%) had been taking BZD for over 6 years, 21 patients (41.2%) between 1 and 6 years, and 8 patients (15.7%) for less than 1 year.

25 patients (50%) began treatment with BZD due to insomnia, with an average consumption period of 5.12 years; 9 patients (18%) were taking BZD due to anxiety, with an average consumption period of 10.67 years, and 5 patients (10%) were depressed, with an average consumption period of 4.15 years. 11 patients (22%) were labelled as “other diagnoses” such us bereavement. divorce, and somatization.

46 patients remembered why BZD had been originally prescribed, with an average consumption period of 7.11 years. In contrast, 5 patients could not recall the reason for their original prescription, with an average consumption period of 4 years.

**Figure 2:**
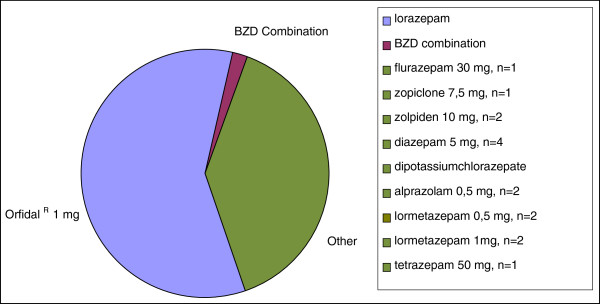
Types of BZD consumed by patients.

Among the patients who agreed to participate in the study 40 (78.4%) were taking short-acting BZD, 8 (15.7%) were using long acting BZD, and 3 (5.9%) took other non-barbiturate hypnotics known as Z-drugs. The BZD most consumed in our study was 1 mg of Lorazepam (58%), equivalent to 10.4 of Diazepam (Figure 2).

The amount of information conveyed by the physician to the patient, when the prescription was first issued, was nil in the case of 31 patients, minimal for 13 patients, and only partial for the remaining 7 patients.

By the end of the intervention program, 80.4% of the patients had stopped their BZD intake and 64.7% maintained abstinence for one year.

In the analysis of the Short-Form Health Survey (SF-12) at baseline the mental component (45.96) consistently achieved a higher score than the physical one (39.60). Both results showed scores below the population mean (50).

Within the group of patients who ceased BZD, comparative analysis of the SF-12 before the intervention and at 24 weeks showed an improvement of 3.3 points (p = 0.02) in the mental component (Table 3). The same comparative analysis of its physical component was made, but no statistically significant differences were observed. Within the group of non-compliance there were no statistically significant improvements in any of the physical or mental components.

**Table 3:** Comparison of quality of life (SF-12), Goldberg, and MOS scales

	**No success (12 pacs)**			**Success (39 pacs)**			
**Scale**	**Mean**	**Desv. Est.**	**p**	**Mean**	**Desv. Est.**	**p**	**Effect size Cohen’s**
SF·12: physical component							
0	41.89	11.62		39.60	8.82		0.25
12 months	49.09	1.63	0.36	39.89	8.36	0.79	1.27
24 months	49.71	2.98	0.58	38.99	7.49	0.52	1.62
SF12: mental component							
0	42.79	16.12		45.96	9.88		−0.28
12 months	48.03	11.17	0.28	48.79	9.74	0.08	−0.08
24 months	40.72	19.63	0.43	49.27	9.73	0.02	−0.69
Goldberg anxiety							
0	3.75	3.19		3.23	2.65		0.19
12 months	0.75	1,50	0,30	2,33	2,31	0,01	−0.75
24 months	2,67	2,52	1,00	2,26	2,42	0,01	0.17
Goldberg depression							
0	2,17	1,85		2,36	2,31		−0.09
12 months	1,00	1,41	0,42	1,62	2,42	0,00	−0.28
24 months	1.00	1,73	0,42	1,55	1,84	0,00	−0.31
Short of Breath Scale							
0	75,00	28,44		82,56	24,36		−0.30
12 months	95,00	10,00	0,42	85,64	20,49	0,32	0.51
24 months	100,00	0,00	0,07	90,26	16,46	0,02	0.69
Sleep Adequacy							
0	40,00	24,12		56,15	27,30		−0.62
12 months	30,00	34,64	0,07	56,41	22,18	0,96	−1.06
24 months	53,33	11,55	0,06	58,46	26,31	0,36	−0.22
Sleep Disturbance Scale							
0	51,88	14,19		55,71	14,78		−0.27
12 months	68,44	10,87	0,19	56,60	13,02	0,73	0.96
24 months	57,92	6,88	0,68	57,31	12,15	0,44	0.06
Sleep Problems Index I							
0	61,39	14,32		62,99	9,88		−0.15
12 months	75,00	15,99	0,46	63,85	6,86	0,60	1.17
24 months	67,78	6,94	0,15	65,47	6,69	0,07	0.35
Sleep Problems Index II							
0	59,35	11,42		60,94	9,34		−0.16
12 months	71,53	12,86	0,40	61,45	5,54	0,83	1.32
24 months	62,04	6,99	0,47	62,68	6,61	0,18	−0.10
Snoring Scale							
0	71,67	35,63		66,15	37,32		0.15
12 months	100,00	0,00	0,18	70,77	35,79	0,24	0.95
24 months	100,00	0,00	0,18	74,87	29,99	0,03	0.97
Somnolence Scale							
0	59,44	17,16		73,50	17,54		−0.82
12 months	70,00	20,73	0,21	72,82	15,09	0,86	−0.17
24 months	64,44	20,37	0,12	76,92	14,25	0,25	−0.80

MOS: Medical Outcomes Study.

SF-12: Short-Form Health Survey.

From analysis of the scales (Table 3) it can be seen that in the MOS sleep scale, those patients taking BZD chronically obtained, at the beginning, a higher score in all parameters of sleep analysis than the population mean. The average hours of sleep was 6.10 (SD 1.46), and 16 patients slept an adequate number of hours (7-8 hours). Within the compliant group results from the same scale showed no difference in most of the components throughout the intervention. Two exceptions were deterioration in snoring perception and an increase in breathing difficulty. Within the non-compliance group there were no statistically significant differences.

Analysis of the Goldberg Depression and Anxiety Scale at the beginning of the program showed an average score compatible with depression but not anxiety (breakpoints are ≥ 4 for the anxiety scale and ≥ 2 for depression). The unsuccessful group showed a lower mean in the depression component at baseline, but without statistically significant differences between the beginning and the end of the study. The same group also showed a higher mean in the anxiety score although still below the anxiety threshold.

## Discussion

The study was designed to show that a BZD withdrawal program, with nursing professionals as the key element, can be applicable within the Spanish Primary Care setting. The following points were considered in developing our program: a) In agreement with many authors, patients should not be forced to cease their treatment [12]; b) The use of complementary medicines as co-adjuvant therapy. Numerous trials have been carried out using complementary medicines to control withdrawal symptoms while the patients are on BZD [13-15]. These trials have highlighted the successful use of Hydroxyzine [16] due to its good tolerance, low risk, and reduced cost; c) Tapered dose. Other studies have also focused on a progressive cessation of BZD [17,18], which reveal a cost-effective alternative for BZD withdrawal; d) Pharmacological support. Although cognitive behavioural therapy is an effective adjuvant therapy in BZD withdrawal programs [19,20] its implementation in daily practice can have significant cost and time implications. Several clinical trials have, therefore, focused instead on providing psychological support to patients during their withdrawal period, based on identifying the reason prescriptions were initiated and maintained, and patients’ information on dependence and withdrawal symptoms [21,22]; and e) The use of Valerian for improving sleep. Whilst Valerian has a subjective improvement on sleep quality it does not reflect an improvement in sleep onset latency [23].

After the level of commitment required to successfully complete the first stage of this programme (i.e. to attend at least six appointments with the nurse and to complete multiple assessment questionnaires) had been explained, 28 of the selected patients did not wish to participate. Most of these patients gave as their reason for refusal the fact of being in active employment and, therefore, limited in terms of time. It should also be noted that the study excluded young patients, which additionally reduced our patient sample. As a result, our sample group consisted predominantly of elderly and retired patients (particularly female pensioners with a low socio-economic level) with concomitant chronic diseases.

Their socio-demographic features matched those of patients defined as being at risk of chronic BZD use [24,25].

Our sample size was large enough to show the beneficial effect of a BZD cessation programme and highlight the difference between the patients’ condition before and after the intervention programme. We recognize, however, that size was the main limitation of the study, and we were unable to obtain statistically significant differences between patients who successfully completed the cessation programme and those who did not. The latter comparison was not, however, a key objective although we consider it could become a useful starting point for further studies.

It should be pointed out that almost 20% of the patients consumed antidepressants, mostly from the Selective Serotonin Reuptake Inhibitor (SSRI) group. This highlights non-compliance with national guidelines based on previous studies [26] which advise limiting the use of BZD to the onset of SSRI treatment. More than twenty years ago it was reported that the benefit of BZD was significantly reduced after fifteen days of use [27]. Yet, as our results confirm, BZD continues to be prescribed without due consideration for this benefit/time restriction.

A comparison revealed that the average daily dose of BZD in the United Kingdom (equivalent to 5 mg of Diazepam) [7] was lower than that in our study in Spain (equivalent to 10 mg of Diazepam). Nevertheless, the percentage of cessation at six months was the same in both countries. Results achieved in withdrawal programmes in both countries were not, therefore, influenced by the initial BZD dosage. In our study, patients taking a higher initial dose of BZD presented more sleeping problems in the baseline test than those taking lower doses; once, however, they had all completed the program, those who had initially consumed more BZD did not score higher on the sleeping problem scale. This finding helped to confirm that the consumption of a higher dose of BZD does not improve sleep quality [7,28]. It is noteworthy, that 10% of the patients who were taking BZD (with its concomitant side effects) [2,29] in excess of four years were unaware of the reason for its indication.

When analysing the SF-12 results before the initial intervention and at 24 weeks, we saw an improvement in the mental component but not in the physical one among the cessation group. These results are similar to those expected as consumption of BZD should not influence physical fitness.

In the analysis of the Goldberg scale, the cessation group showed a statistically significant gradual decrease in both the anxiety and depression components. These results are positive when compared with the study by Curran et al. [7] which, whilst using a different parametric scale (the Geriatric Depression Scale), reported neither an improvement nor a deterioration of these symptoms for the cessation group.

The results of the sleep scale could be interpreted as being due to a heightened awareness of other underlying chronic diseases. In fact, a quarter of the patients had Chronic Obstructive Pulmonary Disease (COPD) and they should not have been taking BZD as a chronic treatment. Discontinuing BZD prevents severe symptoms being masked, and thus permits early action on potential complications. A 64.7% success rate (percentage of BZD cessation cases maintained for one year) shows that, after specific training, nurses in a Primary Care setting can implement a program with the objective of achieving the cessation of BZD intake in volunteer patients, with similar results to studies conducted by other professionals such as physicians or psychologists [7,30,31]. Nevertheless, it must be conceded that our outcome could be partly attributed to an inaccurate diagnosis of insomnia, mistaking this, and the patient’s addiction due to tolerance [32], for physiological sleep disturbances related to age, which are easy to treat through advice and sleep hygiene. Our results may provide another overview [33] of the initial and medium term results of this withdrawal program and represent a starting point for further studies on effectiveness.

## Conclusions

Our withdrawal program, conducted by nurses, was successful in that a period of one year. 2/3 of the patients in our sample ceased taking BZD. These results are similar to studies conducted by other professionals such as physicians or psychologists. Our work confirms the fact that nurses in a Primary Care setting can successfully implement a BZD withdrawal program.

## Abbreviations

BZD: Benzodiazepine;SF-12: Short-Form Health Survey;MOS: Medical Outcomes Study;SSRI: Selective Serotonin Reuptake Inhibitor;COPD: Chronic Obstructive Pulmonary Disease;MAOI: Mono Amin Oxidase Inhibitors

## Competing interests

The authors declare that they have no competing interest.

## Authors’ contributions

CL: Principal investigator, protocol design, seminar speaker, interpretation of the data, writing of the manuscript. XM: Study management, protocol design, data interpretation, writing of the manuscript. BC: Study management, protocol design, patient recruitment. JLV: Statistical analyses, data interpretation, writing of the manuscript. DL: Patient recruitment. ED: Study management, protocol design, data interpretation. All the authors have read and approved the final manuscript.
